# Impact of high- *versus* low-dose neuromuscular blocking agent administration on unplanned 30-day readmission rates in retroperitoneal laparoscopic surgery

**DOI:** 10.1371/journal.pone.0197036

**Published:** 2018-05-23

**Authors:** Martijn Boon, Chris Martini, H. Keri Yang, Shuvayu S. Sen, Rob Bevers, Michiel Warlé, Leon Aarts, Marieke Niesters, Albert Dahan

**Affiliations:** 1 Department of Anesthesiology, Leiden University Medical Center, RC Leiden, The Netherlands; 2 Merck & Co., Center for Observational and Real World Evidence, Merck & Co, Inc., Kenilworth, NJ, United States of America; 3 Department of Urology, Leiden University Medical Center, RC Leiden, The Netherlands; 4 Department of Surgery, Radboud University Medical Center, Nijmegen, The Netherlands; Massachusetts General Hospital, UNITED STATES

## Abstract

Recent data shows that a neuromuscular block (NMB) induced by administration of high doses of rocuronium improves surgical conditions in certain procedures. However, there are limited data on the effect such practices on postoperative outcomes. We performed a retrospective analysis to compare unplanned 30-day readmissions in patients that received high-dose *versus* low-dose rocuronium administration during general anesthesia for laparoscopic retroperitoneal surgery. This retrospective cohort study was performed in the Netherlands in an academic hospital where routine high-dose rocuronium NMB has been practiced since July 2015. Charts of patients receiving anesthesia between January 2014 and December 2016 were searched for surgical cases receiving high-dose rocuronium and matched with respect to procedure, age, sex and ASA classification to patients receiving low-dose rocuronium. The primary post-operative outcome was unplanned 30-day readmission rate. There were 130 patients in each cohort. Patients in the high- and low-dose rocuronium cohorts received 217 ± 49 *versus* 37 ± 5 mg rocuronium, respectively. In the high-dose rocuronium group neuromuscular activity was consistently monitored; matched patients were unreliably monitored. All patients receiving high-dose rocuronium were reversed with sugammadex, while just 33% of matched patients were reversed with sugammadex and 20% with neostigmine; the remaining patients were not reversed. Unplanned 30-day readmission rate was significantly lower in the high-dose compared to the low-dose rocuronium cohort (3.8% *vs*. 12.7%; p = 0.03; odds ratio = 0.33, 95% C.I. 0.12–0.95). This small retrospective study demonstrates a lower incidence of unplanned readmissions within 30-days following laparoscopic retroperitoneal surgery with high-dose relaxant anesthesia and sugammadex reversal in comparison to low-dose relaxant anesthesia. Further prospective studies are needed in larger samples to corroborate our findings and additionally assess the pharmacoeconomics of high-dose relaxant anesthesia taking into account the benefits (reduced readmissions) and harm (cost of relaxants and reversal agents) of such practice.

## Introduction

Typically, general anesthesia is induced by administration of a hypnotic agent to provide loss of consciousness, an opioid analgesic to blunt hemodynamic and stress responses and a muscle relaxant to facilitate tracheal intubation and improve surgical conditions [1,2]. Recently we showed that a neuromuscular block (NMB) induced by the administration of high-dose rocuronium, produces superior surgical conditions compared to the traditionally used NMB induced by low-dose rocuronium in laparoscopic retroperitoneal urologic and bariatric surgery [3,4]. This has been replicated by others in various abdominal and non-abdominal procedures [5–9]. Moreover, a high-dose rocuronium NMB effectively precludes unexpected deterioration of the surgical field at any time [3–9]. Prevention of sudden patient movements is important during critical procedures, such as ophthalmic and neuro-radiologic intervention procedures. Additionally, recent data suggest that high-dose rocuronium NMB may improve pain scores after laparoscopic surgery [4], although this is not a consistent finding [3,10].

Although tightly controlled randomized trials have shown improved subjective scores of surgical conditions when using high-dose rocuronium NMB, there is little evidence that the use of a high-dose rocuronium NMB improves outcome such as reduced postoperative pain scores or less postoperative surgical complications. In addition, the use of muscle relaxants during anesthesia is associated with postoperative residual neuromuscular blockade and respiratory complications [11,12]. Therefore, the intraoperative benefits of high-dose rocuronium NMB during surgery must be weighed against possible postoperative complications. Currently, one possible strategy to prevent residual NMB induced by high-dose rocuronium NMB is reversal with sugammadex, a rocuronium- and vecuronium-specific reversal agent that has been associated in previous studies with a reduction in the incidence of postoperative respiratory complications [13,14]. However, the cost of sugammadex is a consideration and the cost benefit of high-dose rocuronium NMB has therefore been questioned due to uncertain effect on outcomes and high cost of reversal [15].

Given the results of the majority of studies showing improved surgical conditions during high-dose rocuronium NMB, we routinely apply such a NMB in close coordination with our surgical colleagues since July 2015. The high-dose rocuronium NMB is now as part of our clinical practice in a variety of surgical procedures including laparoscopic abdominal procedures, eye surgery and neuroradiological interventions. To understand the utility of high-dose rocuronium anesthesia under “real world” conditions and to address the above-mentioned gap in the evidence on outcomes, we performed a retrospective analysis of chart data to compare high-dose *versus* low-dose rocuronium administration in patients undergoing laparoscopic retroperitoneal surgery. In the analysis we focused on unplanned 30-day readmission rates. Unplanned 30-day readmission is a quality measure of patient care and is costly [16–18]. Previous studies showed that an important factor associated with unplanned readmission is surgical complexity, with infectious complications being the most common indication for readmission [16,19–21]. We hypothesized that the intraoperative advantage created by high-dose rocuronium (*i*.*e*. high-dose rocuronium NMB) may ultimately translate into less unplanned readmissions within the 30 days following the elective surgical procedure. The choice of focusing on laparoscopic retroperitoneal surgery is based on the fact that in our institution the majority of general anesthetics with high-dose relaxant is applied for these procedures as they benefit the most from high-dose rocuronium NMB compared to other laparoscopic procedures [3].

## Methods

High-dose rocuronium NMB was applied using a standard operating procedure that was introduced in July 2015 in our hospital for a series of procedures unless contraindicated (*e*.*g*. allergies to rocuronium or sugammadex, an estimated glomerular filtration rate < 30 mL/min). The high-dose rocuronium NMB is induced by an induction dose of rocuronium (1 mg/kg), followed by a continuous infusion (range 20–50 mg/h) aimed at a post-tetanic count of 1–2 as measured by the TOF CUFF monitor system. After approval of the protocol by the local institutional review board (Commissie Medische Ethiek, Leiden University Medical Center, 2300 RC Leiden, The Netherlands) and registration at clinicaltrials.gov (NCT03174223), fully anonymized data of patients that received anesthesia from January 2014 to December 2016 were retrieved from two electronic medical record databases (Healthcare Information X-change (HIX) and Patient Data Management System (PDMS) both from Chipsoft, The Netherlands). The electronic databases were searched for cases that received general anesthesia with high-dose rocuronium by continuous infusion for any type of elective procedure by using HIX and PDMS specific queries. High-dose rocuronium was defined by a 1 mg/kg bolus dose at induction followed by a continuous infusion. Next, we identified cases that received high-dose rocuronium from July 2015 to December 2016 were matched (one-on-one) for type of surgery, sex, age and American Society of Anesthesiologists (ASA) physical status classification to a case that had received general anesthesia with low-dose rocuronium from January 2014 to July 2015. Low-dose rocuronium was defined by a 0.4 mg/kg bolus dose at induction and absent continuous infusion during surgery. The matching was performed on type of surgery, sex, age (ages could differ by a maximum of 4 years) and ASA status (ASA class 1 and 2 were combined into one group). The research team checked and validated the data manually for consistency and accuracy.

The following data were compared between high- and low dose rocuronium NMB: (1) patient-related data, including age, sex, weight, body mass index (BMI) and ASA physical status classification; (2) data related to anesthesia and surgery, including surgery type, hemodynamics (blood pressure and heart rate; average of values collected during surgery), depth of anesthesia as measured by bispectral index monitoring (average of values collected during surgery), drugs administered and their dose, duration of surgery, duration of anesthesia, postoperative pain scores (measured on an 11-point verbal rating scale (VRS) from 0, no pain to 10, worst imaginable pain); (3) immediate respiratory complications as defined by hypoxemic events in the PACU (SpO_2_ < 90%), bronchospasm, pneumonia during hospital stay; (4) admissions to intensive or intermediate care departments during hospital stay; (4) thirty-day unplanned readmission data (cause of readmission, duration of readmission and relevant patient data of readmitted cases); (5) cost of relaxation and reversal. Pain scores were obtained in the post-anesthesia care unit (PACU) at 15 min intervals and on the ward three-times daily. The pain data were averaged for location (*i*.*e*. average pain data obtained at the PACU and on the ward).

Data were analyzed comparing the high-dose relaxant *versus* low-dose relaxant groups by paired-*t-*test, Mann-Whitney U test or χ^2^ test, depending on the type of data and data distribution. Data are presented as mean ± SD or median or interquartile range (IQR); p-values < 0.05 were considered significant. Data analysis was performed using GraphPad Prism version 7.00 for MAC OS X (GraphPad Software, La Jolla, CA).

The primary outcome of our study was the incidence of unplanned readmission within 30-days of the elective surgical procedure; secondary outcome was cost of relaxation and reversal in the high-and low-dose rocuronium cohorts. Additionally, we compared duration of surgery and anesthesia, and postoperative pain between cohorts.

## Results

### Patients and procedures

During the search period 51,000 anesthetics were administered for various procedures at Leiden University Medical Center. The initial selection procedure resulted in 537 cases that were considered eligible for inclusion in the analysis. High-dose rocuronium anesthesia was administered most frequently to patients undergoing urological procedures, ophthalmologic surgery or surgical renal procedures. During the manual validation process 407 cases were excluded for the following reasons: age < 18 years, lack of surgical procedure code, multiple patient inputs, missing time stamps, absence of reversal dosing information, or procedures other than elective laparoscopic retroperitoneal surgeries (these procedures included open eye surgeries (n = 77), gastrointestinal surgery (n = 14), organ transplantations (n = 15), neurosurgical procedures (n = 4) and gynecological procedures (n = 1)). The final data set consisted of 130 cases that received high-dose rocuronium in patients undergoing elective laparoscopic retroperitoneal surgery. Matching was successful in 100% of cases yielding a total of 130 cases that on average received 217 ± 49 mg rocuronium and 130 matched cases that received 37 ± 5 mg. Patient characteristics and surgical procedures are given in Table 1. Patients in the two cohorts had similar age, ASA classification, weight and body mass index. Due to the predominance of urology in our cohort, males were in the majority (in each cohort 98 men and 32 women). Glomerular filtration rate was above 30 mL/min in all patients receiving high-dose rocuronium and in 125 patients receiving low-dose rocuronium.

**Table 1:** Patient characteristics and retroperitoneal laparoscopic procedures.

	High-dose relaxant	Low-dose relaxant	P-value
**Patient characteristics**			
Number of patients (*n*)	130	130	
Men/women (*n*/*n*)	98/32	98/32	
Age (years) [mean (95% CI)]	60 (58–63)	59 (56–61)	0.46
Weight (kg) [median (IQR)]	81 (73–92)	80 (72–93)	0.65
BMI (kg/m^2^) [median (IQR)]	26 (24–29)	25 (23–29)	0.21
ASA score 1	34%	32%	
ASA score 2	60%	62%	
ASA score 3	6%	6%	
ASA score 4	0%	0%	
ASA score 5	0%	0%	
CKD stage 1–3 (*n*)	130	125	
CKD stage 4 and 5 (*n*)	0	5	
**Laparoscopic retroperitoneal procedures (*n*)**			
Nephrectomy (partial + complete)	56	56	
Retroperitoneal lymph node resection	36	36	
Prostatectomy	24	24	
Pyelumplasty	14	14	

Values are numbers (n), percentages or mean (95% confidence interval (CI)) or median and interquartile range (IQR). CKD chronic kidney disease. CKD stage 1–3: glomerular filtration rate ≥ 30 mL/min; CKD stage 4 and 5; glomerular filtration rate < 30 mL/min.

### Anesthesia and surgery

The two study cohorts differed by a factor of 6 in the amount of rocuronium that was administered (Table 2; S1 Fig). For induction and maintenance all patients received propofol combined with remifentanil, sufentanil or both. More patients in the high-dose relaxant group received remifentanil (65% *vs*. 33%) at on average a 20% higher dose compared to patients in the low-dose relaxant group (p = 0.015, Table 2). Similarly, patients that received sufentanil in the high-dose relaxant groups received on average a 14% higher dose than patients in the low-dose relaxant group (p = 0.025, Table 2).

**Table 2:** Medication administered during anesthesia.

	High dose rocuronium	Low dose rocuronium	
Propofol (induction and maintenance) (*n*)	130	130	
Propofol dose (mg.kg^-1^.min^-1^)	0.13 ± 0.06	0.13 ± 0.05	p = 0.62
Remifentanil (*n*)	71	33	
Remifentanil dose (μg.kg^-1^.min^-1^)	0.16 ± 0.05	0.13 ± 0.04 04	p < 0.001
Sufentanil (*n*)	87	108	
Sufentanil dose (μg/kg)	1.5 ± 0.8	1.3 ± 0.6	p = 0.025
Rocuronium dose (mg)	217 ± 49	37 ± 5	p < 0.0001
Sugammadex (*n*)	130	44	
Sugammadex dose (mg)	267 ± 101 [40–800]	212 ± 55 [100–400]	p < 0.001
Neostigmine (*n*)	-	26	
Neostigmine dose (mg)	-	1.4 ± 0.6	
Atropine (*n*)	-	26	
Atropine dose (mg)	-	0.6 ± 0.2	

Values are number of patients (n) or mean ± SD.

All patients in the study received rocuronium as muscle relaxant. Patients that received high-dose rocuronium were all reversed with sugammadex (dose 267 ± 101 mg), while patients in the low-dose rocuronium group were either not reversed (46%), received sugammadex (34%, dose 212 ± 55 mg, p < 0.001 *vs*. high-dose relaxant group) or received neostigmine (20%, dose 1.4 ± 0.6 mg). Duration of anesthesia, duration of surgery and heart rate values did not differ between cohorts. A small difference in bispectral index (BIS) values was observed with a deeper anesthesia level in patients receiving high-dose than low-dose rocuronium. However, the difference was small (mean difference = 1.5 with 95% confidence interval 0.1–3.0, p = 0.03). Similarly, a small difference in mean arterial pressure was observed between groups (mean difference 5.5 mmHg with 95% confidence interval 3–8 mmHg, p < 0.01). See Tables 2 and 3.

**Table 3:** Measurements during anesthesia.

	High dose rocuronium	Low dose rocuronium	
Duration of surgery (h)	2.4 (1.7–3.1)	2.3 (1.7–3.1)	p = 0.765
Duration of anesthesia (h)	3.1 (2.4–4.1)	3.1 (2.4–4.1)	p = 0.88
Stay in PACU (h)	1.9 (1.4–2.2)	1.7 (1.2–2.2)	p = 0.16
Mean arterial pressure (mmHg)	85 ± 10	80 ± 11	p < 0.01
Heart rate (beats/min)	68 ± 11	69 ± 11	p = 0.48
Bispectral Index	42 ± 5	44 ± 6	p = 0.03
Oxygen saturation (%)	99 ± 1	99 ± 1	p = 0.95
Length of Hospital Stay (d)	2.2 (1.7–3.2)	2.2 (1.3–3.3)	p = 0.52

Values are median (interquartile range) or mean ± SD; PACU = post-anesthesia care unit.

There were no differences in postoperative pain between the two groups in the PACU (median VRS high-dose relaxant: 3.0, with interquartile range (IQR) 1.0–4.7 *vs*. low-dose relaxant: 2.7, IQR 1.0–4.0; p = 0.19) and the ward (median VRS high-dose relaxant: 2.0, IQR 1.0–3.3 *vs*. low-dose relaxant: 2.0, IQR 1.1–3.1 p = 0.89).

Length of hospital stay did not differ between the two cohorts: high-dose rocuronium median duration 2.2 days (IQR 1.7–3.1 days) *vs*. low-dose rocuronium 2.2 (1.3–3.3) days (p = 0.52). In both cohorts there were no immediate postoperative respiratory complications or inadvertent admissions to intensive or intermediate care departments.

### Readmission rates

In the two cohorts, 26 patients were readmitted within 30 days. In seven patients, readmission was pre-planned (reasons for readmission: chemotherapy, learning how to catheterize, screening for kidney transplantation, reassessment of renal function, bisphosphonate treatment) occurring in both cohorts. In 19 (7.3%) patients readmissions was unplanned: 5 (3.8%) in patients receiving high-dose rocuronium and 14 (10.8%) in patients receiving low-dose rocuronium (χ^2^ = 4.6, p = 0.03; odds ratio = 0.33, 95% C.I. 0.12–0.95). In Table 4 the reasons for readmission are given. None of the patients died during readmission. See Table 4 for a specification of the data. In total eight readmissions were related to an infection at the surgical site (1 in patients receiving high-dose rocuronium, 7 in patients receiving low-dose rocuronium).

**Table 4:** 30-day unplanned readmission.

	High dose rocuronium	Low dose rocuronium	P-value
**Unplanned readmissions (95% confidence interval)**	3.8% (1.3–8.8%)	12.7% (6.0–17.4%)	0.03
male/female (*n*/*n*)	4/1	13/1	
Age (years) (median, range)	56 (27–69)	63 (43–75)	0.29
ASA classification (*n*)			
1	2	7	
2	3	7	
3	0	0	
CKD stage 1–3	5	13	
CKD stage 4–5	0	1	
Reasons of readmission	- Abscess in renal bed - Brain metastasis - Enteritis - Lymphocele (n = 2)	- Collapse/hypotension - Enteritis and transient ischemic attack - Intra-abdominal urinary leak - Complicated urinary tract infection (n = 7) - Urinary retention - Lymphocele - Urethra/bladder anastomosis leak - Worsening of renal function (CKD stage 2 to 3)	

CKD chronic kidney disease. CKD stage 1–3: glomerular filtration rate ≥ 30 mL/min; CKD stage 4 and 5; glomerular filtration rate < 30 mL/min.

### Cost

Combined cost of relaxation and reversal was on average $131 (95% confidence interval $115-$147) per patient in the high-dose rocuronium cohort *versus* $34 (95% confidence interval $26-$42) in the low-dose cohort. All of these costs were paid by the hospital. Total reimbursement from reimbursement agencies for readmissions (at the benefit of the hospital) was $116,000 for the 14 patients in the low-dose rocuronium cohort against $42,000 for the 5 patients in the high-dose rocuronium cohort (average reimbursement per patient was similar between the 2 cohorts at approx. $8,300 per patient).

## Discussion

This retrospective matched cohort study presents data of the everyday clinical use of high-dose rocuronium neuromuscular block for elective laparoscopic retroperitoneal surgery in a single tertiary academic hospital in the Netherlands. A total of 130 patients who received high-dose rocuronium anesthesia (and reversal with sugammadex) were compared to 130 matched patients that received low-dose rocuronium anesthesia (to induce a moderate NMB). The main finding of this study is that the administration of high-dose rocuronium is associated with reduced unplanned readmission rates in the thirty days following elective surgery (3.8% *vs*. 12.7%). Furthermore, the application of high-dose rocuronium anesthesia was without inadvertent admissions to intensive or intermediate care departments.

Neuromuscular blocking agents are routinely used in anesthesia to facilitate endotracheal intubation and improve surgical conditions [1,2]. A recent meta-analysis of 12 randomized controlled trials concluded that high-dose rocuronium NMB (causing a so-called deep NMB with PTC 1–3) improves surgical conditions in a variety of procedures as determined by the scoring of the surgical space conditions by the attending surgeon [22]. In most studies included in this review, the applied scoring systems combine the impression of visibility, surgical space, muscle contractions, handling tactics and patient movement into one numerical rating score. In all studies, the effect of the high-dose rocuronium NMB on the score is modest (on average the score improves by about 20%) but considered highly clinically significant by the surgical team [3,4]. Still, the risk of postoperative complications from residual NMB has historically precluded the use of high dose relaxants for many years [11,12]. With the introduction of sugammadex, reversal of high-dose rocuronium NMB is now possible [13,23]. In our institute we now apply high-dose rocuronium anesthesia upon request of surgeons to allow a stable and qualitatively superior surgical space conditions in a variety of procedures.

Our current analysis shows that the both cohorts did not differ in hemodynamics, duration of surgery and anesthesia or immediate postoperative conditions, including postoperative pain. A small difference in depth of anesthesia and mean arterial pressure was observed although we do not consider these differences clinically relevant. We previously identified a small positive effect of high-dose rocuronium NMB on postoperative pain scores following bariatric surgery but not following retroperitoneal urologic procedures [3,4]. The above mentioned meta-analysis of 12 studies also revealed a small advantage of high-dose rocuronium NMB over standard or low-dose rocuronium NMB with an improvement in postoperative numerical pain scores of 0.5 on an 11-point scale [22]. These data suggest that the effect of high-dose rocuronium NMB on pain scores is small. Different procedures may activate specific pathophysiologic pain pathways that interact differently with the high-dose rocuronium NMB. In the current analysis we focus on retroperitoneal surgeries. These procedures are associated with relatively low pain scores and little effect of high-dose rocuronium NMB [3]. Hence, our analysis does not allow definite conclusions regarding the influence of high-dose rocuronium NMB on postoperative pain.

We encountered a reduction in unplanned readmission rates from 12.7% in the low-dose to 3.8% in the high-dose rocuronium cohort (Table 4). Readmissions after surgery are relatively common, with rates ranging from 4–16% after major urologic surgery and 9–20% after colorectal surgery [24,25]. Readmission after surgery represents a major cost for the health care system. For instance, readmission after colorectal surgery costs $9000 per readmission; this annually accounts for $300 million in the US alone [26]. To address and reduce costs, readmission is increasingly being used as a hospital quality indicator by reimbursement agencies. In the US, as a part of the Hospital Readmission Reduction Program, reimbursements may be denied in case of excessive readmission rates [27]. The cost of readmission to reimbursement agencies in our institute is approximately $8,300 per patient. Given the reduction in readmissions observed in patients receiving high-dose rocuronium anesthesia (combined with sugammadex reversal) compared to patients receiving low-dose rocuronium anesthesia, there is a potential financial benefit to the healthcare system that could offset the costs associated with high doses of rocuronium and sugammadex made by the hospital. As this retrospective data may be biased due to changes in surgical and/or anesthesia practice over time, possible inaccuracies in the dataset or its retrieval from HIX or PDMS or other relevant limitations (see below), a prospective randomized pharmacoeconomic trial should be considered to definitely capture relevant cost data.

Known risk factors for readmission include older age, comorbid conditions, length of procedure, surgery complexity, intra- and postoperative complications [16–19,20,21,28]. Due to matching, in our study all relevant factors were comparable at baseline (Tables 1 and 2). We therefore account the application of high-dose rocuronium anesthesia as the most important factor associated with the lower readmission rates. Although our study does not answer the question why better chemical relaxation results in improved surgical outcome, we believe that it is important to discuss possible mechanisms. All procedures were performed in the retroperitoneal space, where working space is very limited. As previously shown, high-dose rocuronium NMB leads to superior scoring of the surgical space conditions in these retroperitoneal surgeries [3,22]. Possibly, the improved surgical conditions in the narrow space of retroperitoneal laparoscopic surgery during high-dose rocuronium NMB contribute to superior technical performance of the surgeon and less trauma to tissue. A recent review supports this hypothesis by concluding that superior technical performance positively affects patient outcome [29]. An important reason for readmission was an infectious complication of the urinary tract (complicated urinary tract infection including pyelonephritis and urosepsis). We hypothesize that urinary tract tissue is particularly susceptible to infection when surgical space conditions are less optimal and/or urinary tract tissue is more frequently or more intensely manipulated (causing micro traumata). Additionally, it is important to realize that NMB agents possess anti-inflammatory properties through actions at the nicotinic acetylcholine receptor α1 [30]. The high-dose rocuronium infusion may therefore have had some tissue protective effect leading to less postoperative complications. Reduced glomerular filtration rate (chronic kidney disease, CKD, stage 4 and 5 with a glomerular filtration rate < 30 mL/min) affects rocuronium clearance. This may be a cause of residual NMB and postoperative complications, especially in those patients that are not or inadequately monitored. However in patients with complications that required readmission just 1 patient receiving low-dose rocuronium and none receiving high-dose rocuronium had CKD stage 5 (Table 4). The reason for readmission in this one particular patient was severe enteritis and a transient ischemic attack. Consequently, we do not think that renal function influenced the outcome of our study. Finally, in other surgery types such as ophthalmology, we did not observe a difference in readmission rates (data not shown). This suggests that the improved outcome in our study is most importantly related to the type and site of surgery.

Our study has various limitations that need to be considered:

1. Since the use of high-dose rocuronium anesthesia is relatively new to our clinical practice, we were able to just include a small number of patients in our analysis. This small sample precluded the analysis of underlying contributing risk factors as a cause of readmission.
2. We remain unaware whether the results of our study are related to the high-dose rocuronium infusion, the reversal with sugammadex or to both. The number of patients that received sugammadex in the low-dose rocuronium cohort was too small to perform a separate analysis.
3. This is a single-center study and generalizability to other centers is likely to be limited. For example, the use of a rocuronium infusion (and reversal with sugammadex) is not standard care in other hospitals in Europe or the US.
4. This is a non-randomized study with a non-contemporaneous control group. We cannot exclude that temporal changes in anesthesia or surgical care may have contributed to the study outcome.
5. More subjects in the high-dose rocuronium group received the opioid remifentanil and irrespective of the type of opioid, the opioid dose was higher in the high-dose rocuronium group. This is a surprising observation that so far remains unexplained. We do not perceive a role for the difference in opioid dose in our study outcome, although it might explain the slightly deeper levels of anesthesia in the high-dose rocuronium cohort (Table 3).
6. Our dataset lacks complete TOF data, as TOF measurements are not automatically recorded in our electronic patient database system. Although neuromuscular monitoring devices are available in all our operating rooms and monitoring is mandatory by local protocols, it cannot be ruled out that part of the patients were in fact inadequately or not monitored, especially in the low dose rocuronium group. This may be a source of residual confounding, however to what extent this influenced our study remains unclear.

Evidently, further prospective randomized controlled trials (with appropriate monitoring) in larger populations are required to determine causal inferences between the relaxant dose and patient outcome and a possible role for the reversal agent on patient outcome.

In conclusion, this small retrospective study presents the first real world data of the effect of a high-dose rocuronium/sugammadex anesthetic technique on postoperative outcome. Our data indicate that the use of a high-dose rocuronium/sugammadex technique is associated with reduced readmission rates in the thirty days following elective laparoscopic retroperitoneal surgery compared to low-dose rocuronium infusions. Given the study limitations, future prospective randomized studies in larger samples are needed to further investigate the benefit of high-dose relaxants on patient outcome. Additionally, the prospective study of the pharmacoeconomic effect of such practice taking into account benefit (reduced readmission rates) and harm (cost of relaxants and reversal agents) is imminently needed.

## Supporting information

S1 File S1 Fig contains the raw data of the study. (XLSX)

S1 Fig Drug doses in the high-dose and low-dose rocuronium cohorts. A. Rocuronium. B. Propofol. C. Remifentanil. D. Sufentanil. E. Sugammadex. F. Neostigmine. Grey dots are individual data. Boxes represent the median and interquartile range, whiskers the range, + the mean value.
(TIFF)
