# Supplementary figures and images for: Impact of high- versus low-dose neuromuscular blocking agent administration on unplanned 30-day readmission rates in retroperitoneal laparoscopic surgery
Source: PLoS One. 2018 May 23;13(5):e0197036. doi: 10.1371/journal.pone.0197036 (PMC5965817; doi:10.1371/journal.pone.0197036)

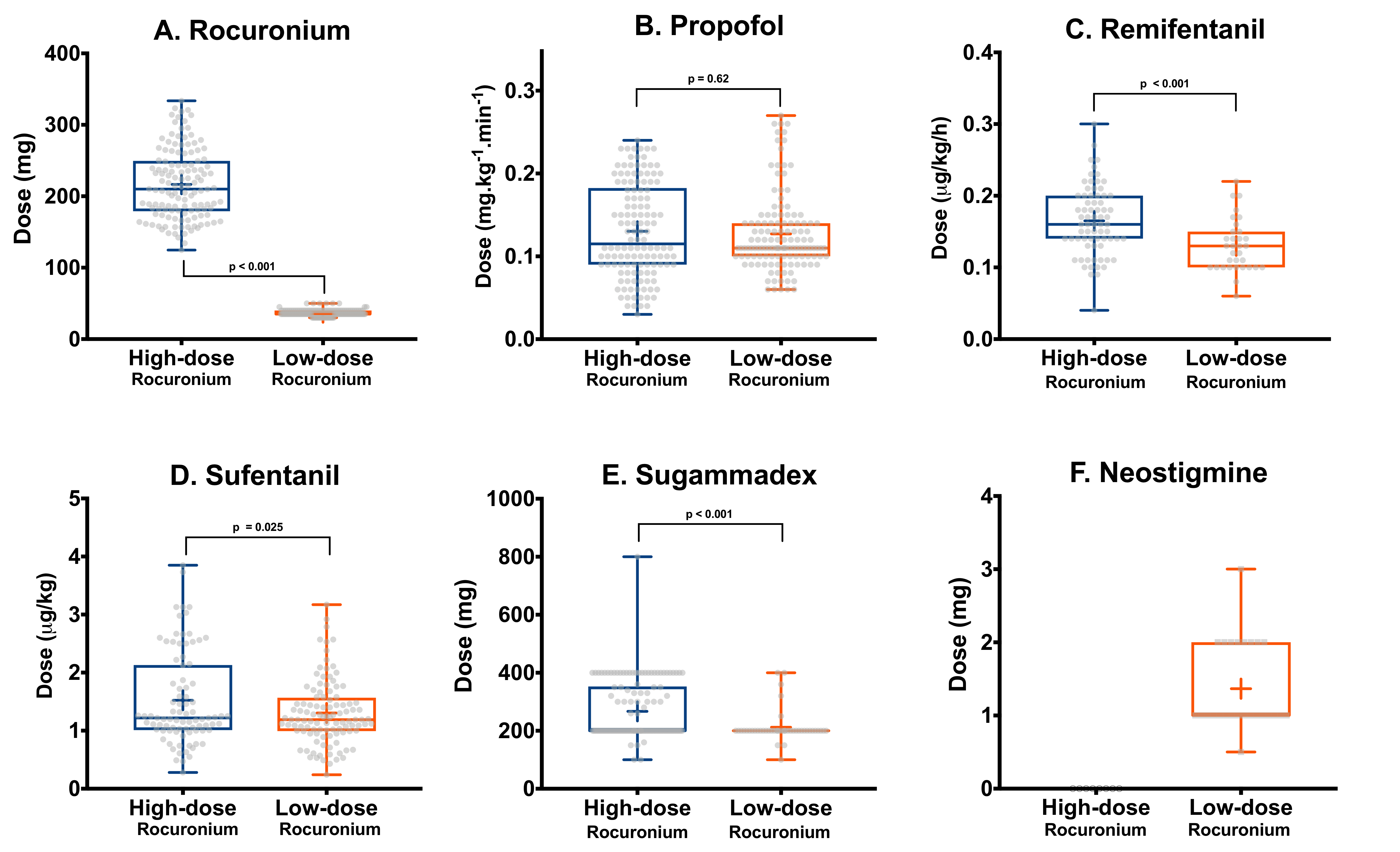

Supplement: S1 Fig — A. Rocuronium. B. Propofol. C. Remifentanil. D. Sufentanil. E. Sugammadex. F. Neostigmine. Grey dots are individual data. Boxes represent the median and interquartile range, whiskers the range, + the mean value. (TIFF) [file pone.0197036.s002.tiff]
